# 1-[(1,3-Dithio­lan-2-yl)meth­yl]-8-nitro-6-propyl-1,2,3,5,6,7-hexa­hydro­imidazo[1,2-*c*]pyrimidine

**DOI:** 10.1107/S1600536810033829

**Published:** 2010-08-28

**Authors:** Dongmei Li, Zhongzhen Tian, Haijun Dong, Gaolei Wang

**Affiliations:** a Shandong Provincial Key Laboratory of Fluorine Chemistry and Chemical Materials, School of Chemistry and Chemical Engineering, University of Jinan, People’s Republic of China; b School of Sciences, University of Jinan, People’s Republic of China

## Abstract

In the title compound, C_13_H_22_N_4_O_2_S_2_, the six-membered ring displays a half-chair conformation. The olefin amine unit is close to being coplanar with the imidazolidine ring (r.m.s. deviation = 0.059 Å). The dithiol­ane ring adopts a twisted conformation. In the crystal, mol­ecules are linked by weak C—H⋯O inter­actions.

## Related literature

For related structures, see Tian *et al.* (2010 ▶); Li *et al.* (2010 ▶). For background to neonicotinoid insecticides, see Mori *et al.* (2001 ▶); Ohno *et al.* (2009 ▶); Jeschke *et al.* (2008 ▶); Kagabu (1997 ▶); Tian *et al.* (2007 ▶).
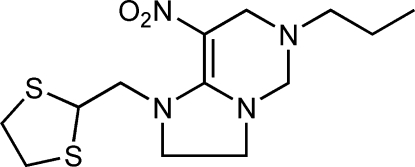


## Experimental

### 

#### Crystal data

C_13_H_22_N_4_O_2_S_2_
*M* *_r_* = 330.47
Monoclinic, 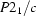

*a* = 11.9680 (3) Å
*b* = 13.6304 (3) Å
*c* = 10.8866 (3) Å
β = 115.465 (3)°
*V* = 1603.38 (8) Å^3^
*Z* = 4
Mo *K*α radiation
μ = 0.34 mm^−1^
*T* = 293 K
0.45 × 0.41 × 0.26 mm

#### Data collection

Bruker APEXII CCD diffractometer
Absorption correction: multi-scan (*SADABS*; Bruker, 2005 ▶) *T*_min_ = 0.917, *T*_max_ = 1.0
13404 measured reflections
3256 independent reflections
2486 reflections with *I* > 2σ(*I*)
*R*_int_ = 0.024

#### Refinement

*R*[*F* ^2^ > 2σ(*F* ^2^)] = 0.033
*wR*(*F* ^2^) = 0.096
*S* = 1.04
3256 reflections
191 parameters
H-atom parameters constrained
Δρ_max_ = 0.22 e Å^−3^
Δρ_min_ = −0.16 e Å^−3^

### 

Data collection: *APEX2* (Bruker, 2005 ▶); cell refinement: *SAINT* (Bruker, 2005 ▶); data reduction: *SAINT*; program(s) used to solve structure: *SIR97* (Altomare *et al.*, 1999 ▶); program(s) used to refine structure: *SHELXL97* (Sheldrick, 2008 ▶); molecular graphics: *SHELXTL* (Sheldrick, 2008 ▶); software used to prepare material for publication: *WinGX* (Farrugia, 1999 ▶).

## Supplementary Material

Crystal structure: contains datablocks I, global. DOI: 10.1107/S1600536810033829/hb5612sup1.cif

Structure factors: contains datablocks I. DOI: 10.1107/S1600536810033829/hb5612Isup2.hkl

Additional supplementary materials:  crystallographic information; 3D view; checkCIF report

## Figures and Tables

**Table 1:** Hydrogen-bond geometry (Å, °)

*D*—H⋯*A*	*D*—H	H⋯*A*	*D*⋯*A*	*D*—H⋯*A*
C3—H3*A*⋯O1^i^	0.97	2.48	3.322 (2)	145
C3—H3*B*⋯O1^ii^	0.97	2.56	3.269 (2)	130
C4—H4*A*⋯O2^i^	0.97	2.52	3.449 (2)	160

Symmetry codes: (i) 
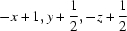
; (ii) 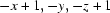.

**Fig. 1.**
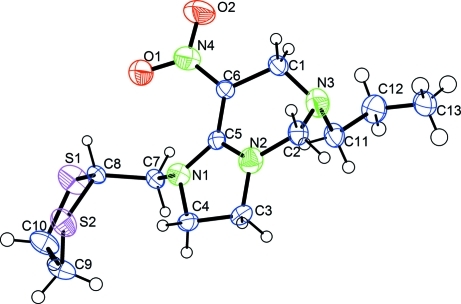
The molecular structure of (I) with displacement ellipsoids drawn at the 30% probability level. The H atoms are shown as spheres of arbitrary size.

## supplementary crystallographic information

### Comment

Neonicotinoid inseticides have become an important chemical class of insecticides (Ohno *et al.*, 2009 and Jeschke *et al.*, 2008). We have synthesized a series of new compounds by introducing sulfur atoms into the lead struture to improve the lipid solubility of neonicotinoids insecticides. in which the title compound exhibited moderate insecticidal activities against pea aphids.

The structure of the title compound is shown in Fig. 1 with the atom-numbering scheme. The title compound is homolog of 1-[(1,3-Dithiolan-2-yl)methyl]-6-methyl- 8-nitro-1,2,3,5,6,7-hexahydroimidazo- [1,2-*c*]pyrimidine (Tian *et al.*, 2010). The six-membered ring displays an half-chair conformation. The olefin-amine moity is close to being coplanar with imidazolidine ring. The dithiolane is in a twisted conformation [C9—C10—S1 = 110.31 (13)° and C9—S2—C8 = 94.61 (8)°]. The packing of the molecules is mainly stabilized by C—H···O interactions (Table 1).

### Experimental

A mixture of 1-((1,3-dithiolan-2-yl)methyl)-2-(nitromethylene)imidazolidine (0.75 mmol), formaldehyde (1.6 mmol, in the form of 35% aqueous solution), and propylamine (0.83 mmol) in ethanol (5 ml) was stirred overnight. The solution thus obtained was concentrated under vacuum and further purified by flash chromatography to give the desired product. Colourless prisms of (I) were obtained by slow evaporation of a solution of dichloromethane and ethyl acetate of the title compound.

### Refinement

All H atoms were placed in their calculated positions and then refined using riding model with C—H = 0.96–0.98 Å, *U*_iso_(H) = 1.2 (1.5 for methyl groups) times *U*_eq_(C).

### Crystal data

**Table:**

C_13_H_22_N_4_O_2_S_2_	*F*(000) = 704
*M**_r_* = 330.47	*D*_x_ = 1.369 Mg m^−^^3^
Monoclinic, *P*2_1_/*c*	Mo *K*α radiation, λ = 0.7107 Å
Hall symbol: -P 2ybc	Cell parameters from 7164 reflections
*a* = 11.9680 (3) Å	θ = 3.3–28.9°
*b* = 13.6304 (3) Å	µ = 0.34 mm^−^^1^
*c* = 10.8866 (3) Å	*T* = 293 K
β = 115.465 (3)°	Prism, colourless
*V* = 1603.38 (8) Å^3^	0.45 × 0.41 × 0.26 mm
*Z* = 4	

### Data collection

**Table:**

Bruker APEXII CCD diffractometer	3256 independent reflections
Radiation source: fine-focus sealed tube	2486 reflections with *I* > 2σ(*I*)
graphite	*R*_int_ = 0.024
Detector resolution: 16.0355 pixels mm^-1^	θ_max_ = 26.4°, θ_min_ = 3.5°
φ and ω scans	*h* = −14→14
Absorption correction: multi-scan (*SADABS*; Bruker, 2005)	*k* = −17→17
*T*_min_ = 0.917, *T*_max_ = 1.0	*l* = −13→13
13404 measured reflections	

### Refinement

**Table:**

Refinement on *F*^2^	Primary atom site location: structure-invariant direct methods
Least-squares matrix: full	Secondary atom site location: difference Fourier map
*R*[*F*^2^ > 2σ(*F*^2^)] = 0.033	Hydrogen site location: inferred from neighbouring sites
*wR*(*F*^2^) = 0.096	H-atom parameters constrained
*S* = 1.04	*w* = 1/[σ^2^(*F*_o_^2^) + (0.0605*P*)^2^] where *P* = (*F*_o_^2^ + 2*F*_c_^2^)/3
3256 reflections	(Δ/σ)_max_ < 0.001
191 parameters	Δρ_max_ = 0.22 e Å^−^^3^
0 restraints	Δρ_min_ = −0.16 e Å^−^^3^

### Special details

**Table:**

Geometry. All e.s.d.'s (except the e.s.d. in the dihedral angle between two l.s. planes) are estimated using the full covariance matrix. The cell e.s.d.'s are taken into account individually in the estimation of e.s.d.'s in distances, angles and torsion angles correlations between e.s.d.'s in cell parameters are only used when they are defined by crystal symmetry. An approximate (isotropic) treatment of cell e.s.d.'s is used for estimating e.s.d.'s involving l.s. planes.
Refinement. Refinement of *F*^2^ against ALL reflections. The weighted *R*-factor *wR* and goodness of fit *S* are based on *F*^2^, conventional *R*-factors *R* are based on *F*, with *F* set to zero for negative *F*^2^. The threshold expression of *F*^2^ > σ(*F*^2^) is used only for calculating *R*-factors(gt) *etc*. and is not relevant to the choice of reflections for refinement. *R*-factors based on *F*^2^ are statistically about twice as large as those based on *F*, and *R*- factors based on ALL data will be even larger.

### Fractional atomic coordinates and isotropic or equivalent isotropic displacement parameters (Å^2^)

**Table:**

	*x*	*y*	*z*	*U*_iso_*/*U*_eq_	
C1	0.21596 (14)	0.03042 (13)	0.24992 (18)	0.0490 (4)	
H1A	0.1584	0.0170	0.1565	0.059*	
H1B	0.1937	−0.0110	0.3083	0.059*	
C2	0.30273 (15)	0.15638 (14)	0.41120 (18)	0.0527 (4)	
H2A	0.2960	0.1151	0.4803	0.063*	
H2B	0.2949	0.2242	0.4336	0.063*	
C3	0.52500 (16)	0.21274 (12)	0.46180 (18)	0.0488 (4)	
H3B	0.5517	0.2262	0.5578	0.059*	
H3A	0.5015	0.2737	0.4112	0.059*	
C4	0.62423 (15)	0.15974 (12)	0.43666 (18)	0.0489 (4)	
H4A	0.6609	0.2028	0.3931	0.059*	
H4B	0.6889	0.1360	0.5214	0.059*	
C5	0.44343 (13)	0.07042 (11)	0.34006 (14)	0.0358 (3)	
C6	0.34568 (13)	0.00437 (12)	0.26942 (15)	0.0392 (4)	
C7	0.58540 (13)	0.05456 (12)	0.23001 (15)	0.0389 (4)	
H7B	0.6083	0.1149	0.1993	0.047*	
H7A	0.5103	0.0303	0.1563	0.047*	
C8	0.68837 (13)	−0.02085 (12)	0.25948 (15)	0.0372 (3)	
H8	0.6626	−0.0827	0.2855	0.045*	
C9	0.88413 (18)	0.07980 (17)	0.2809 (2)	0.0653 (6)	
H9A	0.8400	0.1417	0.2552	0.078*	
H9B	0.9721	0.0936	0.3266	0.078*	
C10	0.85620 (19)	0.01841 (19)	0.1568 (2)	0.0737 (6)	
H10B	0.9206	−0.0304	0.1765	0.088*	
H10A	0.8548	0.0597	0.0836	0.088*	
C11	0.19972 (16)	0.19983 (15)	0.17239 (19)	0.0577 (5)	
H11A	0.2578	0.1771	0.1382	0.069*	
H11B	0.2256	0.2647	0.2106	0.069*	
C12	0.07252 (19)	0.20656 (19)	0.0556 (2)	0.0789 (7)	
H12B	0.0785	0.2407	−0.0195	0.095*	
H12A	0.0422	0.1409	0.0250	0.095*	
C13	−0.01760 (19)	0.25861 (18)	0.0930 (3)	0.0896 (8)	
H13B	−0.0983	0.2561	0.0181	0.134*	
H13C	0.0073	0.3258	0.1140	0.134*	
H13A	−0.0200	0.2276	0.1709	0.134*	
N1	0.55919 (10)	0.07677 (9)	0.34598 (13)	0.0372 (3)	
N2	0.42417 (11)	0.14173 (10)	0.41168 (13)	0.0436 (3)	
N3	0.20390 (12)	0.13306 (11)	0.28063 (14)	0.0472 (4)	
N4	0.36386 (12)	−0.08788 (10)	0.23411 (14)	0.0439 (3)	
O1	0.46924 (10)	−0.12073 (8)	0.25825 (12)	0.0487 (3)	
O2	0.26936 (11)	−0.14243 (10)	0.17994 (15)	0.0678 (4)	
S1	0.70823 (4)	−0.04188 (4)	0.10432 (4)	0.05415 (16)	
S2	0.83629 (3)	0.01316 (4)	0.39184 (4)	0.04712 (15)	

### Atomic displacement parameters (Å^2^)

**Table:**

	*U*^11^	*U*^22^	*U*^33^	*U*^12^	*U*^13^	*U*^23^
C1	0.0408 (9)	0.0547 (11)	0.0542 (11)	0.0003 (8)	0.0230 (8)	0.0050 (8)
C2	0.0557 (10)	0.0621 (11)	0.0483 (10)	0.0097 (9)	0.0299 (8)	0.0015 (9)
C3	0.0617 (10)	0.0354 (8)	0.0463 (9)	−0.0014 (8)	0.0205 (8)	−0.0012 (8)
C4	0.0462 (9)	0.0384 (9)	0.0531 (10)	−0.0057 (7)	0.0128 (8)	−0.0025 (8)
C5	0.0377 (8)	0.0368 (8)	0.0319 (7)	0.0036 (6)	0.0140 (6)	0.0059 (6)
C6	0.0372 (8)	0.0417 (9)	0.0399 (8)	−0.0011 (7)	0.0177 (7)	−0.0014 (7)
C7	0.0335 (8)	0.0478 (9)	0.0336 (8)	0.0014 (7)	0.0128 (6)	0.0087 (7)
C8	0.0336 (7)	0.0456 (9)	0.0331 (7)	−0.0021 (7)	0.0150 (6)	0.0033 (7)
C9	0.0534 (11)	0.0806 (14)	0.0620 (12)	−0.0236 (10)	0.0249 (9)	−0.0013 (11)
C10	0.0649 (13)	0.1042 (17)	0.0659 (13)	−0.0218 (12)	0.0414 (11)	−0.0039 (12)
C11	0.0521 (10)	0.0646 (12)	0.0598 (11)	0.0118 (9)	0.0273 (9)	0.0205 (10)
C12	0.0761 (14)	0.0835 (16)	0.0628 (13)	0.0131 (12)	0.0162 (11)	0.0208 (12)
C13	0.0573 (12)	0.0760 (16)	0.122 (2)	0.0094 (11)	0.0252 (13)	0.0350 (15)
N1	0.0340 (6)	0.0366 (7)	0.0393 (7)	−0.0021 (5)	0.0143 (5)	−0.0013 (6)
N2	0.0453 (7)	0.0432 (8)	0.0427 (8)	0.0025 (6)	0.0194 (6)	−0.0047 (6)
N3	0.0445 (7)	0.0536 (9)	0.0483 (8)	0.0105 (6)	0.0246 (6)	0.0094 (7)
N4	0.0429 (7)	0.0457 (8)	0.0447 (8)	−0.0073 (6)	0.0203 (6)	−0.0049 (6)
O1	0.0441 (6)	0.0443 (6)	0.0623 (8)	−0.0003 (5)	0.0273 (5)	−0.0079 (6)
O2	0.0505 (7)	0.0591 (8)	0.0897 (10)	−0.0204 (6)	0.0263 (7)	−0.0248 (7)
S1	0.0480 (3)	0.0784 (4)	0.0390 (2)	−0.0091 (2)	0.02147 (19)	−0.0101 (2)
S2	0.0341 (2)	0.0642 (3)	0.0365 (2)	0.00106 (18)	0.00896 (16)	0.00165 (19)

### Geometric parameters (Å, °)

**Table:**

C1—H1A	0.9700	C8—H8	0.9800
C1—H1B	0.9700	C8—S1	1.8268 (15)
C1—C6	1.516 (2)	C8—S2	1.7976 (15)
C1—N3	1.460 (2)	C9—H9A	0.9700
C2—H2A	0.9700	C9—H9B	0.9700
C2—H2B	0.9700	C9—C10	1.499 (3)
C2—N2	1.465 (2)	C9—S2	1.791 (2)
C2—N3	1.440 (2)	C10—H10B	0.9700
C3—H3B	0.9700	C10—H10A	0.9700
C3—H3A	0.9700	C10—S1	1.8078 (19)
C3—C4	1.512 (2)	C11—H11A	0.9700
C3—N2	1.458 (2)	C11—H11B	0.9700
C4—H4A	0.9700	C11—C12	1.509 (3)
C4—H4B	0.9700	C11—N3	1.473 (2)
C4—N1	1.483 (2)	C12—H12B	0.9700
C5—C6	1.413 (2)	C12—H12A	0.9700
C5—N1	1.3617 (18)	C12—C13	1.486 (3)
C5—N2	1.3269 (19)	C13—H13B	0.9600
C6—N4	1.359 (2)	C13—H13C	0.9600
C7—H7B	0.9700	C13—H13A	0.9600
C7—H7A	0.9700	N4—O1	1.2550 (16)
C7—C8	1.529 (2)	N4—O2	1.2674 (16)
C7—N1	1.4576 (19)		
			
C1—N3—C11	112.44 (14)	C12—C13—H13A	109.5
H1A—C1—H1B	107.8	H12B—C12—H12A	107.8
C2—N3—C1	108.50 (13)	C13—C12—C11	112.7 (2)
C2—N3—C11	112.60 (14)	C13—C12—H12B	109.0
H2A—C2—H2B	108.0	C13—C12—H12A	109.0
C3—C4—H4A	110.8	H13B—C13—H13C	109.5
C3—C4—H4B	110.8	H13B—C13—H13A	109.5
C3—N2—C2	124.84 (14)	H13C—C13—H13A	109.5
H3B—C3—H3A	109.3	N1—C4—C3	104.87 (12)
C4—C3—H3B	111.4	N1—C4—H4A	110.8
C4—C3—H3A	111.4	N1—C4—H4B	110.8
H4A—C4—H4B	108.8	N1—C5—C6	130.82 (14)
C5—C6—C1	119.04 (14)	N1—C7—H7B	108.7
C5—N1—C4	108.19 (12)	N1—C7—H7A	108.7
C5—N1—C7	122.63 (12)	N1—C7—C8	114.33 (12)
C5—N2—C2	121.37 (13)	N2—C2—H2A	109.3
C5—N2—C3	112.42 (13)	N2—C2—H2B	109.3
C6—C1—H1A	109.0	N2—C3—H3B	111.4
C6—C1—H1B	109.0	N2—C3—H3A	111.4
C7—C8—H8	108.3	N2—C3—C4	101.72 (13)
C7—C8—S1	108.97 (10)	N2—C5—C6	118.30 (13)
C7—C8—S2	114.96 (11)	N2—C5—N1	110.84 (13)
C7—N1—C4	119.23 (13)	N3—C1—H1A	109.0
H7B—C7—H7A	107.6	N3—C1—H1B	109.0
C8—C7—H7B	108.7	N3—C1—C6	112.87 (13)
C8—C7—H7A	108.7	N3—C2—H2A	109.3
C9—C10—H10B	109.6	N3—C2—H2B	109.3
C9—C10—H10A	109.6	N3—C2—N2	111.46 (13)
C9—C10—S1	110.31 (13)	N3—C11—H11A	109.0
C9—S2—C8	94.61 (8)	N3—C11—H11B	109.0
H9A—C9—H9B	108.4	N3—C11—C12	112.74 (16)
C10—C9—H9A	110.1	N4—C6—C1	117.12 (13)
C10—C9—H9B	110.1	N4—C6—C5	123.19 (13)
C10—C9—S2	108.21 (15)	O1—N4—C6	122.64 (13)
C10—S1—C8	97.79 (9)	O1—N4—O2	120.23 (13)
H10B—C10—H10A	108.1	O2—N4—C6	117.06 (13)
C11—C12—H12B	109.0	S1—C8—H8	108.3
C11—C12—H12A	109.0	S1—C10—H10B	109.6
H11A—C11—H11B	107.8	S1—C10—H10A	109.6
C12—C11—H11A	109.0	S2—C8—H8	108.3
C12—C11—H11B	109.0	S2—C8—S1	107.87 (8)
C12—C13—H13B	109.5	S2—C9—H9A	110.1
C12—C13—H13C	109.5	S2—C9—H9B	110.1
			
C1—C6—N4—O1	−172.73 (14)	N1—C5—C6—C1	−163.47 (15)
C1—C6—N4—O2	4.3 (2)	N1—C5—C6—N4	26.1 (3)
C3—C4—N1—C5	−10.33 (17)	N1—C5—N2—C2	174.29 (13)
C3—C4—N1—C7	136.21 (14)	N1—C5—N2—C3	7.03 (18)
C4—C3—N2—C2	−179.65 (14)	N1—C7—C8—S1	179.15 (10)
C4—C3—N2—C5	−12.91 (17)	N1—C7—C8—S2	−59.67 (16)
C5—C6—N4—O1	−2.1 (2)	N2—C2—N3—C1	59.98 (18)
C5—C6—N4—O2	174.92 (14)	N2—C2—N3—C11	−65.14 (19)
C6—C1—N3—C2	−49.56 (18)	N2—C3—C4—N1	13.31 (16)
C6—C1—N3—C11	75.65 (17)	N2—C5—C6—C1	14.4 (2)
C6—C5—N1—C4	−179.54 (15)	N2—C5—C6—N4	−156.09 (15)
C6—C5—N1—C7	35.3 (2)	N2—C5—N1—C4	2.50 (17)
C6—C5—N2—C2	−4.0 (2)	N2—C5—N1—C7	−142.66 (14)
C6—C5—N2—C3	−171.22 (13)	N3—C1—C6—C5	13.5 (2)
C7—C8—S1—C10	109.21 (13)	N3—C1—C6—N4	−175.50 (14)
C7—C8—S2—C9	−86.58 (13)	N3—C2—N2—C3	131.22 (16)
C8—C7—N1—C4	91.93 (16)	N3—C2—N2—C5	−34.4 (2)
C8—C7—N1—C5	−126.53 (15)	N3—C11—C12—C13	69.8 (2)
C9—C10—S1—C8	−14.29 (18)	S1—C8—S2—C9	35.20 (11)
C10—C9—S2—C8	−46.45 (16)	S2—C8—S1—C10	−16.21 (11)
C12—C11—N3—C1	82.0 (2)	S2—C9—C10—S1	40.3 (2)
C12—C11—N3—C2	−155.08 (17)		

### Hydrogen-bond geometry (Å, °)

**Table:**

*D*—H···*A*	*D*—H	H···*A*	*D*···*A*	*D*—H···*A*
C3—H3A···O1^i^	0.97	2.48	3.322 (2)	145
C3—H3B···O1^ii^	0.97	2.56	3.269 (2)	130
C4—H4A···O2^i^	0.97	2.52	3.449 (2)	160

Symmetry codes: (i) −*x*+1, *y*+1/2, −*z*+1/2; (ii) −*x*+1, −*y*, −*z*+1.
